# The State of the Art of Material Jetting—A Critical Review

**DOI:** 10.3390/polym13162829

**Published:** 2021-08-23

**Authors:** Orhan Gülcan, Kadir Günaydın, Aykut Tamer

**Affiliations:** 1 General Electric Aviation, Gebze 41400, Kocaeli, Turkey; 2 Department of Mechanical Engineering, Imperial College London, London SW7 2AZ, UK; a.tamer@imperial.ac.uk

**Keywords:** tray location, build direction, surface finish, matte, glossy

## Abstract

Material jetting (MJ) technology is an additive manufacturing method that selectively cures liquid photopolymer to build functional parts. The use of MJ technology has increased in popularity and been adapted by different industries, ranging from biomedicine and dentistry to manufacturing and aviation, thanks to its advantages in printing parts with high dimensional accuracy and low surface roughness. To better understand the MJ technology, it is essential to address the capabilities, applications and the usage areas of MJ. Additionally, the comparison of MJ with alternative methods and its limitations need to be explained. Moreover, the parameters influencing the dimensional accuracy and mechanical properties of MJ printed parts should be stated. This paper aims to review these critical aspects of MJ manufacturing altogether to provide an overall insight into the state of the art of MJ.

## 1. Introduction

The demand for complex parts is steadily increasing in different industries (especially in aerospace, automotive and biomedical industries) to manufacture lighter parts with higher stiffness, higher strength and lower cost. Thanks to the recent advances in additive manufacturing (AM) technologies, engineers have more freedom to design and produce complex parts which were more difficult if not impossible to manufacture with conventional means [1]. The main difference in AM from conventional, subtractive manufacturing methods is that it is based on a layer-by-layer manufacturing which results in a reduced low buy to fly ratio (the ratio of weight of raw material to weight of the final part) [2,3].

**Figure 1:**
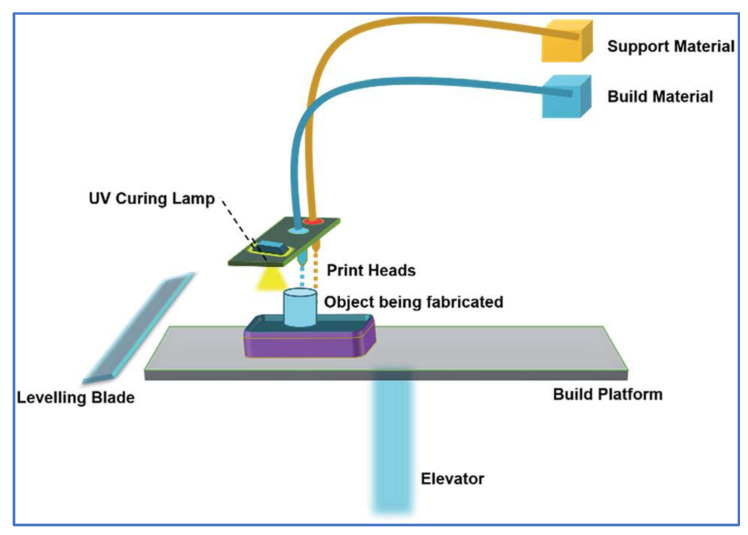
A schematic representation of MJ [14].

According to “The American Society for Testing and Materials (ASTM) Committee F42 on Additive Manufacturing Technologies” AM technologies can be classified as: powder bed fusion, material jetting, vat photopolymerization, directed energy deposition, material extrusion, binder jetting and sheet lamination [4]. The two different terms are utilized to refer to material jetting processes synonymously. These names are used due to secured naming rights of the material jetting printer manufacturers Stratasys (PolyJet) and 3DSystems (MultiJet). The technology was first developed by Objet Geometries Ltd. in 2000 and was acquired later by Stratasys in 2012 [5]. According to ISO/ASTM 52900: 2015 standard, “droplets of feedstock material are selectively deposited” in MJ technology [6]. Although the MJ printer design varies slightly from manufacturer to manufacturer, a general schematic representation of MJ can be seen in Figure 1. In MJ, air-excluding tanks are used to store photopolymer materials and these are deposited as droplets forming a very thin layer on the build platform after heating photopolymer in the transmission line in which photopolymer is transmitted from tank to nozzle [7]. Ultraviolet (UV) light is emitted onto the molten material on the build platform for curing. In this photopolymerization/photo-curing process, a light source of a specific wavelength is used to cure monomers/oligomers in the liquid state [8]. Unlike the wavelength of lamps used by SLA (355 nm) and DLP (405 nm), the wavelength of the light source in MJ can theoretically be unrestricted [9], but, practically, a light source of a wavelength between 190 and 400 nm is used [10,11]. After curing a layer, the build platform is lowered at a level of certain layer thickness amount and new liquid material is jetted onto the previous layer. After curing each successive layer, a full-scale part is completely built [12]. Since liquid or molten material is used in MJ, a gel-like support structure is needed, especially in overhang regions. These support structures are removed from the part using different methods: sonication in a bath of sodium hydroxide solution, heating or using a high-pressure water jet [13].

The MJ technology is a prominent additive manufacturing method in the polymer printing field due its advantages in comparison with other polymer printing techniques. Technology allows us to adapt thin layer thicknesses which allows for printing high-quality parts and less evident staircase effects and thin wall features [8]. The layer thicknesses can be as low as 16 µm [14]. The low surface roughness texture is another advantage which is one of the major problems for additive manufacturing technologies. There are two surface finish options in MJ technology: matte and glossy. In matte the setting, the whole part is covered with support material. In the glossy setting, only structurally needed areas are supported and the model is exposed to air during curing. After printing, supported areas will be matte and unsupported areas will be glossy. Furthermore, no post-processes are required for MJ technology, and parts are usable in the as-built condition after separating them from build platform and support removal processes. Rather than printing parts directly on substrate, a photopolymer resin can be printed onto substrate as a printing bed [15]. Due to easy detachment of photopolymer, separating printed parts from the built plate is possible with a hand tool by applying less amount of force. In addition, different materials such as PLA, ABS, polyamide and their combinations can be combined in a single in MJ technology, called the multi-material approach [14]. The multi-material approach can be utilized to produce composite parts for specific purposes. Lastly, MJ printers have a closed ambient chamber for production which prevents undesirable effects of draught or dirt and can be used in offices and homes. 

In this study, the state of the art of MJ technology was reviewed. To better understand the MJ technology, its capabilities, applications and usage areas were addressed. Additionally, MJ was compared with other technologies and its limitations were examined. Moreover, the parameters influencing the dimensional accuracy and mechanical properties of MJ printed parts were investigated. To the authors’ knowledge, no other review has addressed these aspects of MJ in the literature. Section 2 presents process and materials where polymeric composites are investigated and the effect of tray location, build orientation and surface finish setting on dimensional accuracy, surface roughness and mechanical properties of MJ printed parts are reviewed. In this section, MJ capability and performance of MJ printed parts and comparison of MJ technology with other technologies are also investigated. Section 3 reports the application of MJ technology in different industries. The paper is concluded by a summary of the findings. 

## 2. Process and Materials

### 2.1. Materials

In MJ, thermoplastic, thermosetting and elastomeric polymers with different mechanical properties are used. Pilipović et al. compared mechanical properties of FullCure, VeroBlue and VeroBlack materials in MJ. They stated that FullCure material has the maximum flexural and tensile strength, followed by VeroBlue and VeroBlack. They also mentioned that FullCure material resulted in the lowest surface roughness [16]. O’Neill et al. compared mechanical properties of different types of materials in MJ. They stated that elastic modulus, strain at fracture, maximum compressive strength and yield strength of MED610 is higher than Vero White and RGD525. It was found that RGD 525 has the lowest and MED610 has the highest wear rate [13]. Kaweesa and Meisel designed fatigue test bars in which the middle sections have a material gradient transition region with continuous gradients and stepwise gradient types of functionally graded material interface designs with two materials: VeroCyan and TangoBlackPlus. They found that stepwise gradient specimens with a longer flexible material region showed interfacial failure and higher fatigue life than continuous gradient specimens and fatigue life increased with a decrease in material gradient transition lengths [17].

### 2.2. Polymeric Composites via MJ

The improvement in AM enables new ideas to produce innovative parts to fulfill mechanical needs. One of these ideas is the production of composite materials, which was inspired by natural composites, with a mixture of soft–hard materials to create new materials with better mechanical properties [18]. The combination of two different materials exhibiting different hardness specifications creates unique properties such as improved flexibility and hardness [19,20]. The benefit of AM methods for the production of these complex structures is relatively less time needed and cost in comparison to conventional techniques. These composite materials are generally utilized in applications of specially designed structures such as hierarchical structures, including honeycombs, lattice structures and foams for the purpose of energy absorption [21,22,23]. MJ is one of the most convenient methods for composite material production. Sugawaneswaran et al. suggested a novel methodology for fabrication of a randomly oriented plastic reinforced composite structure with elastomer matrix phase. The random distribution of reinforcing materials was determined in a CAD model. As a result, the composite exhibited 22% more stiffness and 10% more elongation in comparison with constituent materials, while the orientation of reinforcements did not inflences the stress–strain curves considerably [24,25]. One of the composite structures that can be fabricated with MJ is interpenetrating phase composites (IPCs). An IPC is made of lattice-based solid sheets embedded in a soft matrix. Dalag et al. investigated the compression behavior of triply periodic minimal surface (TPMS) reinforced IPCs. It was concluded that an increase in reinforcement structure volume fraction of 5% to 20% affects the mechanical behavior of the IPC. Moreover, with the increase in the reinforcement structure volume fraction, ultimate compression strength and yield strength rise considerably. During the deformation of the IPCs, local bucklings and debonding failure mechanisms were experienced [26]. As for the dimensional accuracy of 3D printed microcomposites via MJ, Tee et al. [27] suggested that geometric resolution is convenient whenever it is greater than 500 µm. Additionally, in this study, the mechanical behaviors of 3D printed polymeric microcomposites with different compositions and arrangements of reinforced particles are investigated. As a result, it is shown that orientations and reinforced particle geometries dominate the stiffness of the composites under compressive loads. However, the composite specimen tensile test results showed that build orientation is insignificant for strength whenever the reinforced particle volume fraction is kept at a level of 5%. In addition, it was stated that the composition and reinforced particle arrangement considerably influences the mechanical behavior. In particular, the hardness of the reinforced particles has an effect on the failure mechanism of the polymeric composite structures. Even though the use of hard particles is able to increase the strength, soft particles serve as crack initiators.

### 2.3. The Effect of Parameters

Dimensional accuracy, surface roughness and mechanical properties of MJ printed parts are crucial to obtain correct and reliable results after measurements and proper functioning in the final assembly. Tray location, layer thickness, build orientation, surface finish, material type and post-processing are the most important parameters that affect dimensional accuracy, surface roughness and mechanical properties of MJ manufactured parts [28,29].

In the literature, the percentage or level of impact of these parameters on dimensional accuracy, surface roughness and mechanical properties has been investigated. Related to surface roughness, Kechagias et al. investigated the effect of layer thickness, surface finish setting and scale factor on the surface roughness of MJ printed parts. They stated that a smaller layer thickness (16 µm) and glossy surface finish setting gives the best results in terms of surface roughness. They also indicated that scale factor was not a dominant factor for surface roughness [30]. Aslani et al. investigated the effect of layer thickness, surface finish setting (matte or glossy) and build scale on surface roughness of MJ printed parts. They observed that the surface finish setting has the greatest effect on surface roughness with a contribution rate of 95%. On the contrary, the layer thickness and the build scale have very little effect on surface roughness with a contribution rate of less than 3% [31]. 

In MJ, dimensional accuracy of printed parts depends mostly on polymerization speed and photocurable resin material viscosity [32]. Kechagias et al. investigated the effect of layer thickness, surface finish setting (matte or glossy) and model scale factor on dimensional accuracy of internal and external features produced by MJ. Their results showed that layer thickness and model scale factor were the two main factors affecting dimensional accuracy of internal features. For external features, all factors were equally important, although layer thickness has higher importance in the X and Y direction and the model scale factor has higher importance in the Z direction [33]. Aslani et al. investigated the effect of layer thickness, surface finish setting and scale on dimensional accuracy of MJ 3D printed parts using the grey Taguchi method. They stated that scale had the largest impact with contribution of 66.54%, followed by build style (contribution = 33.16%) and layer thickness (contribution = 0.3%) [34].

Pugalendhi et al. investigated the effect of finish option, material, thickness and shape on build time, specimen height and number of layers in MJ printed parts. They used Taguchi analysis to perform DOE and ANOVA to determine the % contribution of each factor to the results. They suggested that for all measured values, thickness was the most dominant factor [35].

#### 2.3.1. Tray Location

**Figure 2:**
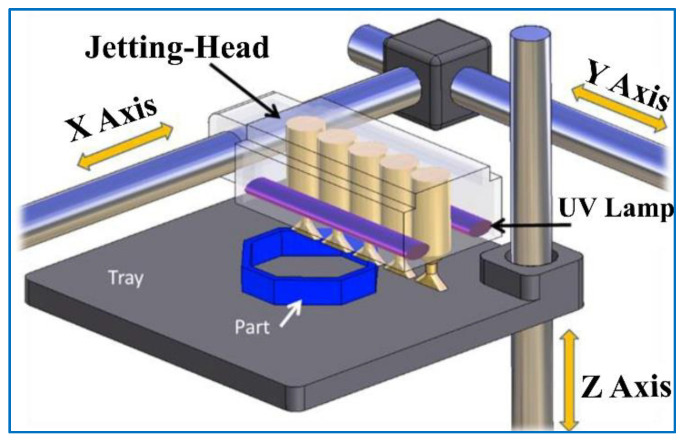
X, Y and Z axis in MJ [36].

In MJ, tray location can be defined in terms of the X, Y and Z axis. The jetting head moves along the X axis and along the transverse Y axis along which jetting orifices are located in parallel. After each layer, the build plate moves along the Z axis (Figure 2) [36]. Locating the part along the X or Y axis of the build tray in MJ has a considerable effect on the mechanical properties and surface roughness of final parts. As stated by Barclift et al., the distribution of the same parts on the build platform can affect mechanical properties due to over curing of some parts in different locations while other parts are being cured. They also stated that decreasing the part spacing increased the part strength [37]. Pilipović et al. stated that dimensional accuracy and repeatability on the X and Y axis is better than it is for the Z axis based on distance measurements in MJ technology [8]. Cazon et al. stated that the best roughness results were obtained when standard tensile specimens (BS EN ISO 527-2:1996) were placed close to the XY plane (in which the first letter designates the specimen’s main axis and the second letter is the minor axis), in other words, parts printed along the X axis gave the best results in terms of stiffness [29]. Gay et al. stated that part spacing on the X axis and surface roughness of parts had no significant influence on mechanical properties but part spacing on the Y axis has a considerable effect on properties. Therefore, the authors suggested placing the parts on the Y axis as close as possible to obtain better mechanical properties. They also stated that orientation of the part affected the mechanical properties slightly and maximum properties were observed when the parts were located parallel to the coordinate axis [36]. Beltrán et al. investigated the effect of orientation, location and size of the part within the build platform on dimensional and geometrical accuracy of cylindrical features. They stated that part orientation and part size have the most influence and part location within the build platform has a relatively lower influence on accuracy of these parts [38].

#### 2.3.2. Build Orientation

The build orientation of parts with respect to the build tray affects surface roughness, dimensional accuracy and mechanical properties. To obtain better values of these characteristics, it is important to know their effect on the properties of the final part. Kumar and Kumar stated that the surface roughness generally increased as build orientation increased until 90° (build orientation with respect to the build direction z) in MJ printed parts [39]. Kumar and Kumar investigated the effect of finish type, local surface orientation and layer thickness on surface roughness of FullCure 720 and VeroBlue 840 printed parts by MJ. They stated that the major factors affecting surface roughness were surface orientation and finish type, and maximum surface roughness was obtained for the 90^o^ surface angle [40]. Kechagias and Stavropoulos reported that when the angle of sloped surfaces of specimens was increased, surface roughness values were also increased. The best and worst values of surface roughness were achieved when the angle of sloped surfaces was zero and 90°, respectively [41]. Khoshkhoo et al. investigated the effect of build orientation and printing direction on asymmetry, rough surface, stair-stepping effects and traces of flowed material along the surface. They suggested that for achieving better surface finish, design fidelity and dimensional accuracy, the build platform can be tilted and printing parameters (build orientation, support strategy) can be changed [42]. Vidakis et al. investigated the effect of sloped surface angle of the part on surface roughness of MJ printed parts. They used 0, 15, 30, 45, 60, 75 and 90° slope values and the X and Y direction as experimental inputs. Results showed that parts produced in the X direction showed better surface roughness when the sloped surface angle was below 45° and, after that value, parts produced in the Y direction had lower surface roughness values [43].

Kent et al. investigated the effect of build orientation on surface roughness and dimensional accuracy of MJ printed parts with four different materials. They found that build orientation has a very high impact on these measurements, but the results were not fully consistent with each other [32]. Haghighi et al. stated that a horizontal build resulted in better dimensional accuracy than vertical builds [44]. Kitsakis et al. investigated the tolerance values in the X, Y and Z direction of MJ printed parts. They reported that accuracy on the Z axis is smaller than on the X and Y axis [45,46].

Kesy and Kotlinski investigated the effect of build orientation on mechanical properties of MJ manufactured parts. In different parts built in different orientations, they found considerable changes in mechanical properties and attributed these changes to variations in the amount of UV energy that reaches the different zones on the build platform for each part [47]. Das et al. studied the effect of build orientation on tensile strength of MJ printed parts. They built the specimens in flat and inclined conditions and X, Y and 45° angle directions. They suggested that specimens built in the X direction and flat conditions showed the highest tensile properties and specimens built in the Y direction and inclined condition showed the lowest tensile properties [48]. Tomar et al. stated that building direction plays an important role in tensile properties of MJ printed parts. Their experimental study revealed that samples produced parallel to the XY plane showed higher values of yield strength, breaking stress and strain at fracture than samples produced parallel to the Z direction [49].

The glass transition temperature is one of the most important properties of polymeric materials and this property is relatively unknown for MJ printed parts. Sanders et al. investigated the effect of build orientation and layer thickness on glass transition temperature in MJ and stated that parts printed in the X direction and with larger layer thickness values demonstrated higher glass transition temperature values [50]. 

Blanco et al. investigated the effect of build direction on the relaxation modulus of MJ printed parts. They concluded that parts built at a 0° slope angle had the highest relaxation modulus; it decreased progressively with a 60°–75° slope angle, then it increased up to a 90° slope angle. According to the authors, a shielding effect from UV curing by the support material could be the main cause of the phenomenon [51]. Reichl et al. looked into the viscoelastic properties of MJ printed parts characterized by a complex modulus which depends on frequency and temperature. They stated that build orientation (vertical or horizontal) had no effect on the complex modulus results [52].

#### 2.3.3. Surface Roughness Options

As mentioned in Introduction, there are two surface finish options in MJ: matte and glossy. The difference between these two settings is that, in the matte setting, the whole part is covered with support material. On the other hand, only structurally needed areas are supported and the model is exposed to air during the curing phase in the glossy setting. Yap et al. stated that when the parts were printed along the Z axis and in the matte setting, higher dimensional accuracy was obtained. On the other hand, the glossy setting reduces the need for support and cost [53]. In MJ technology, there are also two printing modes: high speed and high quality. Pugalendhi et al. investigated the mechanical properties of MJ printed parts from VeroBlue material. They stated that the high-speed printing mode is superior in terms of tensile strength, elongation at break, flexural strength and shore hardness when compared to high-quality printing mode. They also suggested that a glossy finish had lower peaks and valleys and resulted in better surface finish than matte finish options [54]. Pugalendhi et al. compared mechanical properties of VeroWhitePlus and VeroClear and stated that for both materials, glossy finish specimens give better results when compared to matte finish specimens [55]. Cazon et al. stated that the glossy setting gives better surface roughness than the matte setting [29]. Kampker et al. expressed that surface finish has a significant effect on the tensile strength and glossy surface finish parts have higher tensile strength values [56]. Moore et al. presented that glossy finish parts have higher fatigue life when compared to matte finish parts in MJ due to the lower surface roughness obtained with the glossy setting [57]. 

### 2.4. MJ Capability and Performance of MJ Printed Parts

For an effective design, dimensional accuracy and process capability of related technology are paramount. MJ technology is capable of manufacturing dimensionally accurate parts. It can be used in mass production in a short period of time with tolerance values matching IT10 grade for linear dimensions on the Z axis and ISO 286 IT9 grade for radial dimensions [58]. MJ is capable of manufacturing thin walls with a tolerance value of 25–50 μm [12]. By proper selection of parameters, dimensional accuracy tolerances can be lowered to 15 μm [38]. Silva et al. stated that microfeatures larger than 423 µm can be successfully built with MJ and large distortions or printing failures were observed below this value [59]. However, it has some limitations. Holes with diameters smaller than 0.5 mm cannot be horizontally or vertically printed and those with diameters of nearly 1 mm may have some circularity deviations in MJ technology [60]. Tee et al. stated that when parts are printed in multiple materials, MJ is capable of printing microcomposites as small as 62.5 μm but these small parts have high dimensional variations (20% to 75% dimensional variation was observed for parts with length, height and diameter features of between 62.5 μm and 250 μm) [27].

For optimal printing, the liquid material must have enough viscosity, which is generally achieved by heating the material up to 30-70 °C [61]. Yap et al. stated that thin wall features need to be oriented 0° along the Y direction (see Figure 2 for the convention of the directions) and greater than 0.4 mm for a successful build and best accuracy in width and height [53].

Wear performance of MJ printed parts has also been studied. Dangnan et al. investigated the wear and friction mechanism of MJ printed ABS and Verogray polymers. They applied different contact loads (1, 5 and 10N) and stated that in MJ parts, the wear rate depends on the applied load and surface orientation to the sliding direction. For both parallel and perpendicular orientations, the highest coefficient of friction was observed under a 1N load [62]. It was stated that with plastic reinforcements, the elastic modulus of elastomeric parts produced by MJ can be increased by 6.79%–21.03% [24,25].

In the literature, different layer thicknesses and main and support materials have been used in different MJ machines. Some of these studies are summarized in Table 1.

### 2.5. Comparisons with Other Technologies

Polymeric materials can be manufactured using different techniques in addition to MJ, as follows: selective laser sintering (SLS), fused deposition modeling (FDM or fused filament fabrication (FFF)), three-dimensional printing (3DP), binder jetting (BJ), stereolithography (SLA), color-jet printing (CJP), digital light processing (DLP) and laminated object manufacturing (LOM). These technologies were investigated and compared in different aspects in the literature. 

Ramola et al. stated that MJ technology produces 3D models with higher accuracy than SLS or 3DP [83]. Ibrahim et al. investigated the dimensional accuracy of MJ, 3DP and SLS printed mandibular anatomy parts, which showed that SLS parts had a lower dimensional error (1.79%) than MJ (2.14%) and 3DP (3.14%) parts but MJ printed parts had the highest accuracy [84]. Salmi et al. compared dimensional accuracy of SLS, 3DP and MJ printed skull models and stated that the MJ method resulted in the lowest dimensional error (0.18% ± 0.12% for first measurement and 0.18% ± 0.13% for second measurement) [85]. MJ technology also gives rise to less dimensional variance than SLS technology due to the laser dispersion in the build plate [86].

It was asserted in the literature that MJ technology has higher resolution than FDM which may result in the existence of coarse weld lines between successive layers when FDM is used [87]. Lee et al. compared the surface roughness of replica teeth produced by FDM and MJ technology. They stated that MJ resulted in smoother surfaces than FDM due to lower layer thickness values (MJ: 0.016 mm, FDM: 0.330 mm) [82]. Camardella et al. investigated the dimensional accuracy of dental models made with SLA and MJ techniques and reported that all the models manufactured by MJ were accurate [73]. Maurya et al. compared FDM and MJ technology in terms of form error (flatness, roundness and cylindricity), dimensional accuracy, surface roughness, tolerance grade and cost analysis for an automotive part (engine connecting rod). They recorded that MJ printed parts have a lower percentage error along the XY plane, lower average percentage error in circular dimensions, lower form error and lower surface roughness but higher cost than FDM printed parts [76]. 

Kim et al. compared SLA, FDM, MJ, SLS, 3DP and LOM technologies in terms of dimensional accuracy, mechanical properties, surface roughness, printing speed and cost. They noted that MJ technology is advantageous in terms of tensile strength at room temperature [88]. Manoharan et al. compared SLA, MJ, SLS and 3DP in terms of surface finish, dimensional accuracy, materials and printing time for the production of sports footwear. They concluded that MJ showed the best dimensional accuracy, good surface finish, reasonable supported materials and short printing time [89]. Li et al. compared FDM, SLA and MJ technologies in terms of surface roughness, part cost, sustainability and human perception of surface texture and material colors. They concluded that MJ printed parts had the lowest surface roughness but the highest environmental impact and cost [90]. Queral et al. compared dimensional accuracy of SLS, SLA, FDM and MJ manufactured coil winding structures. They stated that high-quality MJ and FDM achieved 0.1% minimum dimensional accuracy for these parts [91]. Tan et al. compared three different AM techniques (MJ, FDM and SLS) in terms of dimensional accuracy. They showed that MJ resulted in the most accurate final part, whereas FDM resulted in the greatest dimensional deviation from the requirements [92]. 

**Figure 3:**
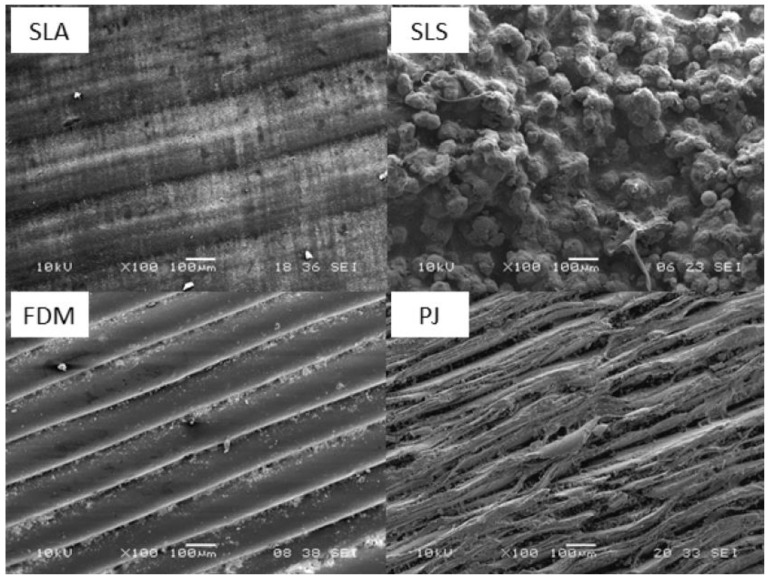
Printed surfaces for SLA, SLS, FDM and MJ samples [96].

One of the ongoing problems for additive manufacturing that needs to be overcome is the dimensional accuracy. In the comparison studies, it was found that MJ technology gives higher dimensional accuracy when printing dental models than SLA, DLP and FFF techniques [71]. Hong et al. compared three different AM techniques in terms of dimensional accuracy of thyroid cancer phantom parts: FDM, CJP and MJ. They stated that MJ gave the best results in terms of dimensional accuracy and clinical demands, but its cost was relatively high [93]. Chen et al. compared reproducibility, dimensional accuracy and dimensional stability of surgical templates produced by three different AM techniques: SLA, MJ and direct metal printing. They concluded that MJ printed templates had the highest accuracy and reproducibility, but their accuracy deteriorated after 1 month of storage [74]. Khaledi et al. compared three different production techniques in metal copings pattern fabrication: milling, SLA and MJ. They stated that the MJ method has a smaller marginal discrepancy than the SLA and milling techniques, meaning that MJ gives the highest accuracy [94]. Dizon et al. compared dimensional accuracy of injected parts from polymer molds produced by SLA, MJ and FDM technologies. They stated that the surface finish of SLA and MJ printed parts was excellent and dimensional accuracy of injected parts from MJ printed molds was higher than from SLA printed molds [77]. Msallem et al. compared the dimensional accuracies of anatomic mandibular models printed with five different AM methods: SLS, BJ, FFF, MJ and SLA. They stated that overall trueness analyses were carried out for SLS, BJ, FFF, MJ and SLA with decreasing trueness [88]. Park et al. compared dimensional variation in dental casts produced by FDM, DLP, SLA and MJ. They stated that casts printed by FDM and DLP showed contraction behavior, whereas casts printed by MJ and SLA showed expansion behavior [95]. Eliasova et al. investigated four different AM techniques in terms of surface roughness and dimensional accuracy. They concluded that MJ samples had fibrous structures and showed higher surface roughness as compared to other techniques. They also specified that surface roughness over the build direction is much higher than that over the perpendicular one for MJ samples (Figure 3) [96]. Budzik et al. compared car mirror holder parts manufactured by MJ, FDM and DLP in terms of visual control, application of a caliper and application of a contactless optical system. They stated that the MJ method was the most precise technique [97]. Wesemann et al. compared the accuracy of occlusal splints produced by SLA, DLP and MJ and concluded that SLA showed the smallest manufacturing deviations [98].

## 3. Applications

### 3.1. Medical Applications

**Figure 4:**
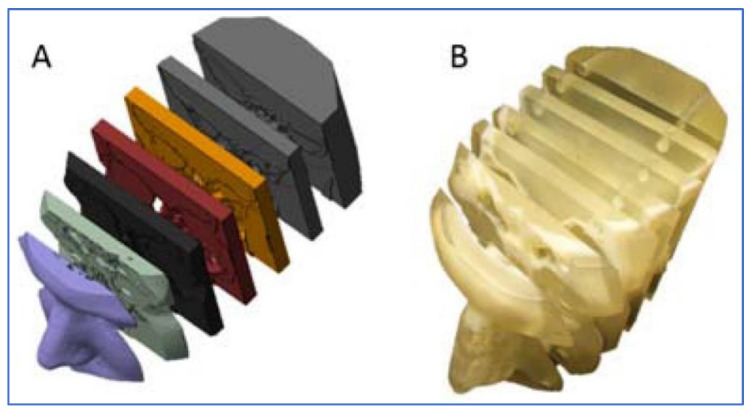
(**A**) CT scan slices of patient nasal cavity, (**B**) MJ printed model [102].

Due to the aforementioned advantages, MJ technology is utilized in different industries for different purposes. In the biomedical industry, MJ is exploited for printing hand protheses by using FullCure 720 material [99]. Manufacturing surgical training models [100] and printing multi-color bone models can also be achieved, which can potentially be used as anatomy teaching tools for specific diseases [101]. For example, printing human nasal sinus anatomy was demonstrated to teach different patients about their medical conditions and surgical treatment options (Figure 4) [102]. In one of the studies, Khalid et al. compared mechanical properties of three different types of materials in MJ: VeroWhitePlus (RGD835), TangoBlackPlus (FLX980) and RigidLightGrey25 (RGD8510-DM) and stated that these materials can be employed in pediatric head impact scenario investigations [103]. 

In dentistry, MJ is used for printing implant surgical templates, mouthpiece fixation instruments and implant guide production. Kim et al. produced maxilla and mandible implant guides with PolyJet, SLA and MultiJet printing (MJP) and stated that PolyJet parts had the lowest dimensional variations [63]. Herschdorfer et al. used PolyJet, SLA and MJP to produce implant surgical templates and stated that no significant effect was found in the accuracy of templates between the three AM processes [64]. Kitamori et al. printed mouthpiece fixation instruments used by head and neck radiotherapy patients by MJ. They replicated the mouthpieces by applying computed tomography to skull bones and produced them by MJ with MED610 material and stated that the templates can successfully be implemented in dosimetry [104]. Anunmana et al. produced implant guides with MJ, DLP and SLA and stated that MJ printed samples showed the highest 3D dimensional accuracy at entry point and apex [65]. Etajuri et al. produced implant guides by applying computed tomography to sheep mandibles with MJ. They stated that the dimensional variations are within acceptable limits of 2 mm [105]. 

### 3.2. Mechanical Applications

**Figure 5:**
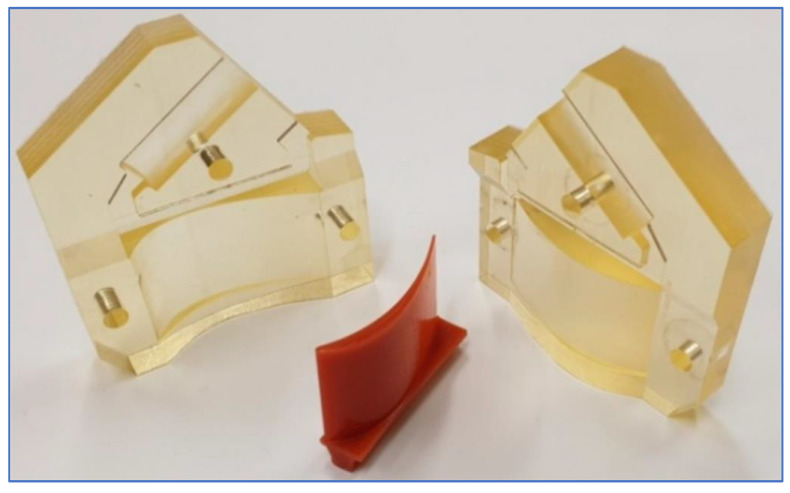
Wax turbine blade from MJ printed molds [106].

In the manufacturing industry, MJ is employed to manufacture plastic injection molds (Figure 5) [66,106] provide final parts ready to validate a product [107] and in tooling. Since MJ inserts have a smoother surface finish than direct metal laser sintering (DMLS) inserts, they can be utilized without any polishing [108].

MJ was used to produce intricate 3D pentamode structures from FullCure850 VeroGray with minimum and maximum ligament thicknesses of 0.71 mm and 1.32 mm, respectively [109]. Honeycomb, re-entrant and auxetic structures was also produced from FullCure850 VeroGray material with MJ technology for crashbox applications [110]. MJ was used to produce dual-material auxetic metamaterials (DMAMs) to increase the mechanical properties and stability of metamaterials. For this purpose, DMAMs were produced by MJ with an accuracy of 0.1 mm from stiff and ductile materials and it was stated that the deformation behavior and Poisson’s ratio of DMAMs in the elastic region were controlled and buckling in stiff regions was prevented [111]. MJ was also used in biomedical applications for control and actuation of robots and robot arms by using metamaterials from rubber-like materials. In one of the studies, it was stated that an inchworm-type soft robot for crawling through channels was produced by MJ and showed good stability and resilience [112].

### 3.3. Acoustic Applications

MJ technology is capable of producing parts with different materials and fillers. One of the applications based on multi-material and filler usage in MJ is producing parts by adding ceramic and metal materials to increase the acoustic ability of the metamaterials [113,114,115]. In one of the studies, special cellular thin-walled specimens based on resonant-type coupled tubes with open-end faces were produced by MJ from VeroWhite 830 material for acoustic applications where the frequency band ranged from 300 to 600 Hz and it was stated that MJ printed parts showed two times higher acoustic power absorption capability than a standard absorber of the same size where a mineral wool layer was covered by a perforated panel [116]. In another study, a sandwich panel with an MJ printed polymer core, unidirectional carbon-fiber composite front and back face sheets and translucent epoxy DP 190 to provide bonding between core and sheets were used. It was stated that this sandwich panel configuration increased the damping performance by nearly 25% without any optimization and it was also proposed that this configuration was a good candidate for pressurized airplane fuselage load-carrying flexural structures [117].

### 3.4. Electronics Applications

In electronics applications, MJ is also utilized due to its unique properties and ability to modify MJ printers to obtain needed structures. Jabari et al. developed and characterized a drop-on-demand piezoelectric–pneumatic material jetting (PPMJ) additive manufacturing process for the aim of printing graphene-based nanocomposites. The developed method exhibited a production rate 10 times faster than current extrusion techniques, in which printing speed reaches about 500 mm/s. As well as the production rate, this technology provides low resistive parts that can compete with other previously reported extrusion-based graphene structure printing methods [118]. Zhang et al. developed a polyimide film for MJ to create an insulating bridge for a circuit. As a result, the produced polyimide film showed a permittivity level of 3.41 and around 500 °C degradation temperature, which are comparable values to commercially polyimide films [119]. Moreover, MJ technology was exploited in the production of low-voltage polymer field-effect transistors [120], and all-polymer capacitors [121]. 

### 3.5. Multi-Material Applications

Multi-material additive manufacturing is a promising research topic that provides both geometrical complexity and material flexibility in one structure. Multi-material production with MJ requires additional print heads to disperse different materials for curing. Local changes in the material in a structure cause increased tensile strength, biocompability, flexibility and visuality with different colors and opacities. Thus, beyond aesthetic uses, a multi-material product is generally used to increase the energy absorption capabilities of lattice structures. Wang et al. produced multi-material auxetic lattice structures for increasing the energy absorption of a structure. Stiff materials were utilized to produce ligaments that are parallel to the compression direction; however, joints in which premature failures occur were fabricated using flexible materials [111]. Moreover, a similar method was also applied to another auxetic lattice structure with the same purpose [122]. As a result, the multi-material production capability of MJ provides design flexibility for energy absorption of structures.

### 3.6. Other Applications

In the aviation industry, MJ can be used in rapid manufacturing and be used to test various wing prototype designs for unmanned aerial vehicle (UAV) applications [4] and to produce wing structures with different types of lattice designs to achieve lightweight aircrafts [123].

MJ technology appeared in fashion industries for its multi-material production ability. For instance, a highly textured cape and skirt in Iris van Herpen’s VOLTAGE collection were MJ printed [124]. Moreover, as an unexpected application area, musical instrument components [67] were manufactured via MJ, and biocompatible plastic toys [68] were also produced. Other interesting purposes for MJ technology include production of bellow actuators [125,126], microfluid mixing devices [127] and devices with integrated porous structures which are used for colorimetric detection of iron in natural waters and soil [69]. In addition, the technology was also adapted to design and fabricate acoustic metamaterial samples with high sound absorption efficiency. Vdovin et al. compared the sound absorption capability of standard absorbers and MJ printed absorbers. They used an Objet Eden 350 PolyJet machine and FullCure 720 and VeroWhite 830 materials. Their results revealed that MJ printed absorbers have a higher sound absorption capability than standard ones due to 3D printing without distortion of the intended geometry. Additionally, additional UV polymerization after the build process reduced residual stresses in the printed samples [116].

Additive manufacturing is associated with the production of complex arbitrary structures such as lattice structures. MJ technology is also utilized in the production of lattice geometries to be used in different types of testing instead of selective laser melting (SLM) technology which has been intensively used in lattice specimen production but the cost of which is much higher than for polymer-based parts [128]. In one study, MJ technology was used to evaluate the compressive behavior of triply periodic minimum surface lattice structures [129]. To overcome the limitation, the technology was used to produced special honeycomb structures in which a solid side wall of the traditional honeycomb structure can be replaced by a porous side wall, which resulted in improved stiffness and strength [130]. Composite polymer parts including ferromagnetic reinforcing particles can also be achieved by developing MJ technology [131].

## 4. Summary

This review focused on state of art of MJ printing technology, and its advantages over other technologies and applications. Today, MJ technology is used in very different industries due to its advantages in producing parts in a relatively short period of time. A detailed literature review showed that MJ technology results in higher dimensional accuracy and better surface roughness values than other polymeric material printing technologies (FDM, SLA, etc.). However, more improvements can still be made by changing the control variables. As consistently reported in the literature, tray location, post-processing, material type, layer thickness, surface finish and build orientation have considerable effects on mechanical properties, surface roughness and dimensional accuracy of the final part. For surface roughness, it was revealed that the surface finish setting has the most effect, whereas for dimensional accuracy; however, layer thickness and model scale are the secondary factors that affect the surface roughness. For better surface roughness values, glossy finish settings need to be chosen during printing. Studies also showed that parts’ distribution on the tray affects mechanical properties and for higher strength results, part spacing needs to be decreased. Build orientation also has considerable influence on the characteristics of the final part and it was stated in the literature that when build orientation increases, surface roughness increases and dimensional accuracy and strength decrease. In the design approach, multi-material prints can be achieved for better or arbitrary mechanical performances. Additionally, obtaining different colors in one build enables better visual designs for different fields in which human perception is necessary.

Based on the findings in the literature, the following research gaps were identified:

- Industrial applications of MJ technology still need further investigation. Especially in the aviation industry, MJ was used only in small prototype wing applications. Its potential for aircraft modification purposes where mock-ups are needed for proper installation is worth investigating.
- MJ technology produces high-quality parts but for higher quality applications, some post-processing techniques need to be applied. Post-processing of MJ printed parts needs further investigation.
- Design engineers need some guidelines for proper designs to be manufactured. For this reason, designs for MJ manufacturing need further investigation.
- Dimensional variations in any production method are very important for proper installation. The producibility of MJ printed parts has been evaluated in the literature but, especially for parts used in aviation and automotive industries, producibility and dimensional variations still needs more attention.

## Figures and Tables

**Table 1:** Materials, machines and layer thicknesses used in the literature.

Main Material	Support Material	Machine	Layer Thickness	Reference
VeroBlack	FullCure 705	Objet Connex 350	-	[8]
VeroWhite MED610 RGD 525	FullCure 705	Objet 260 Connex1	-	[13]
VeroMagenta	-	Objet 500 Connex3	0.03 mm	[63]
VeroClear	-	-	15 μm	[64]
MED610	-	Objet Eden 260VS	16.5 μm	[65]
FullCure 720, VeroWhite, VeroBlue	-	Objet Eden 260	-	[66]
VeroWhite	-	Objet 500	16 μm	[67]
VeroWhite, FullCure 720, ABS-like	-	Objet J750	14–27 μm	[68]
VeroClear	SUP707	Objet Eden 260VS	-	[69]
VeroBlue	FullCure 705	Objet Eden 350	16 μm	[58]
VeroWhite	FullCure 705	Objet 30	28 μm	[38]
FLX935, VeroMagenta	SUP706B	Objet J750	-	[27]
VeroClear, VeroWhitePlus	FullCure 705	Objet 260 Connex2	-	[55]
VeroWhitePlus	FullCure 705	Objet 30	16 μm	[70]
-	-	Objet Eden 260VS	16 μm	[71]
VeroClear	SUP707	Objet Eden 260VS	-	[72]
FullCure 720	-	Objet Eden 260VS	16 μm	[73]
MED610	-	Objet Eden 260VS	-	[74]
RGD240	FullCure 705	Objet 30	28 μm	[36]
TangoBlackPlus VeroWhitePlus	-	Objet Connex 350	30 μm	[57]
FullCure 720	FullCure 705	Objet Eden 350	16 μm	[75]
RGD840	FullCure 705	Objet 30	28 μm	[76]
RGD515	-	Objet 350	16 μm	[77]
FullCure 720	-	Objet Eden 250	16 μm and 30 μm	[31,33]
RGD720	SUP706	Objet 30	-	[78]
Digital ABS Ivory, VeroGray, RGD720 and Rigur	-	Connex2 Objet 500	-	[56]
VeroClear	-	Objet 30 Prime	16–28 μm	[79]
VeroBlackPlus	SUP706B	Objet J750	27 μm	[50]
VeroWhite, RDG525, MED610	-	Objet 260 Connex1	-	[32]
Agilus30 and VeroWhite	SUP706	Objet 500 Connex3	-	[80]
VeroWhitePlus	FullCure 705	Objet 260 Connex	-	[48]
VeroClear	-	Objet 30 Prime	28 μm	[44]
FullCure 720	-	Objet Eden 250	16, 30 μm	[30]
FullCure 720	-	Objet Eden 250	16, 30 μm	[34]
RGD240	FullCure 705	Object 30	28 μm	[51]
FullCure 720, VeroWhite	FullCure 705	Objet Connex 350	32 μm	[81]
VeroWhitePlus	-	Object 30	16 μm	[82]

## Data Availability

The data presented in this study are available on request from the corresponding author.
